# Effect of exposure to deltamethrin on the bufadienolide profiles in *Bufo bufo gargarizans* venom determined by ultra-performance liquid chromatography-triple quadrupole mass spectrometry

**DOI:** 10.1039/c8ra07871h

**Published:** 2019-01-09

**Authors:** Jing Zhou, Huacong Zhao, Luyao Chen, Xiaowei Xing, Tingmei Lv, Xinyi Yang, Qinan Wu, Jinao Duan, Hongyue Ma

**Affiliations:** Jiangsu Collaborative Innovation Center of Chinese Medicinal Resources Industrialization, Jiangsu Key Laboratory for High Technology Research of TCM Formulae, College of Pharmacy, Nanjing University of Chinese Medicine Nanjing 210023 China zhoujing_nj@126.com hongyuema@126.com +86-2585811625

## Abstract

The population of *Bufo bufo gargarizans* Cantor in China has been alarmingly declining due to environmental pollution. Deltamethrin is a pyrethroid pesticide frequently used in agriculture and much of its residues are present in crops, soil and water. Deltamethrin has been shown to have toxicity to toads. Herein, we assumed that deltamethrin contamination might influence the biosynthesis of toxic substances present in toad venom. Bufadienolides present in venom are the toad's chemical defense and highly toxic to predators, and they are important for the survival of toad species against predators. In this study, we determined the contents of bufadienolides in toad venom using a HPLC-triple quadrupole mass spectrometer to evaluate the change in bufadienolide profiles in toad venom before and after cutaneous exposure to deltamethrin. The results indicated that toads exposed to high concentration of deltamethrin survive the least, do not exuviate, and their movements are stiff. Furthermore, it was observed that high level of deltamethrin contamination induces a marked decrease in the levels of toxic bufadienolides in toad venom. These changes in the toxin profiles could lead to the compromised chemical defense of toad, leading to more susceptible to attack by predators. This is the first study to report that environmental contaminants (pesticides) can influence the toad's toxic profiles, suggesting one factor contributing to the decline in the population of *B. bufo gargarizans* Cantor.

## Abstract 2

Deltamethrin contamination could influence the bufadienolide profile in toads.

## Graphical abstract

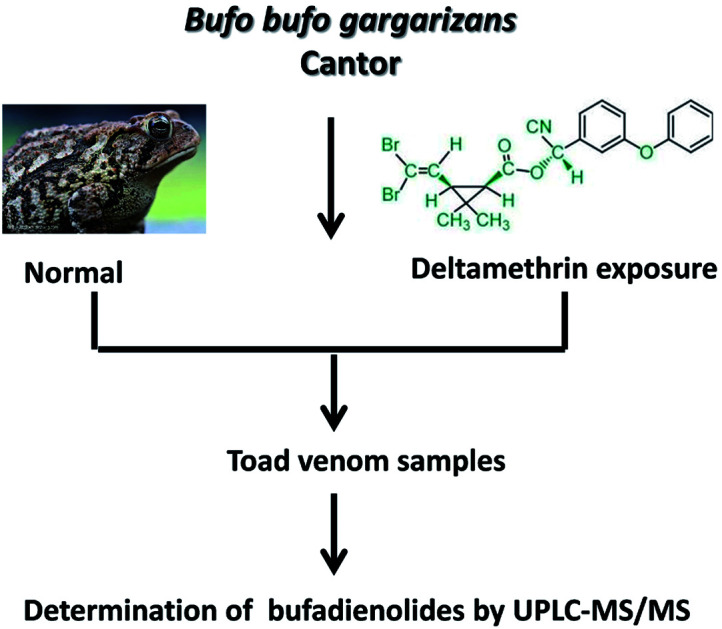

## 1. Introduction

The decline and loss of amphibian populations have become a global problem with complex local causes.^1–4^ One of these causes is environmental toxicants that act directly by killing animals or indirectly by impairing reproduction, reducing growth rates, disrupting normal development and reproduction, disrupting the endocrine system, or increasing susceptibility to disease by immunosuppression or inhibition of the immune system.^4^ Most environmental toxicants are emitted by industrial activities, wastewater, agriculture, the transport sector, and incineration. Pollutants, *e.g.*, pesticides, industrial sewage and heavy metals, are ultimately discharged into rivers along with the current.^5^ Exposure to persistent environmental pollutants is a significant factor causing physiological disorders.^6^

**Fig. 1:**
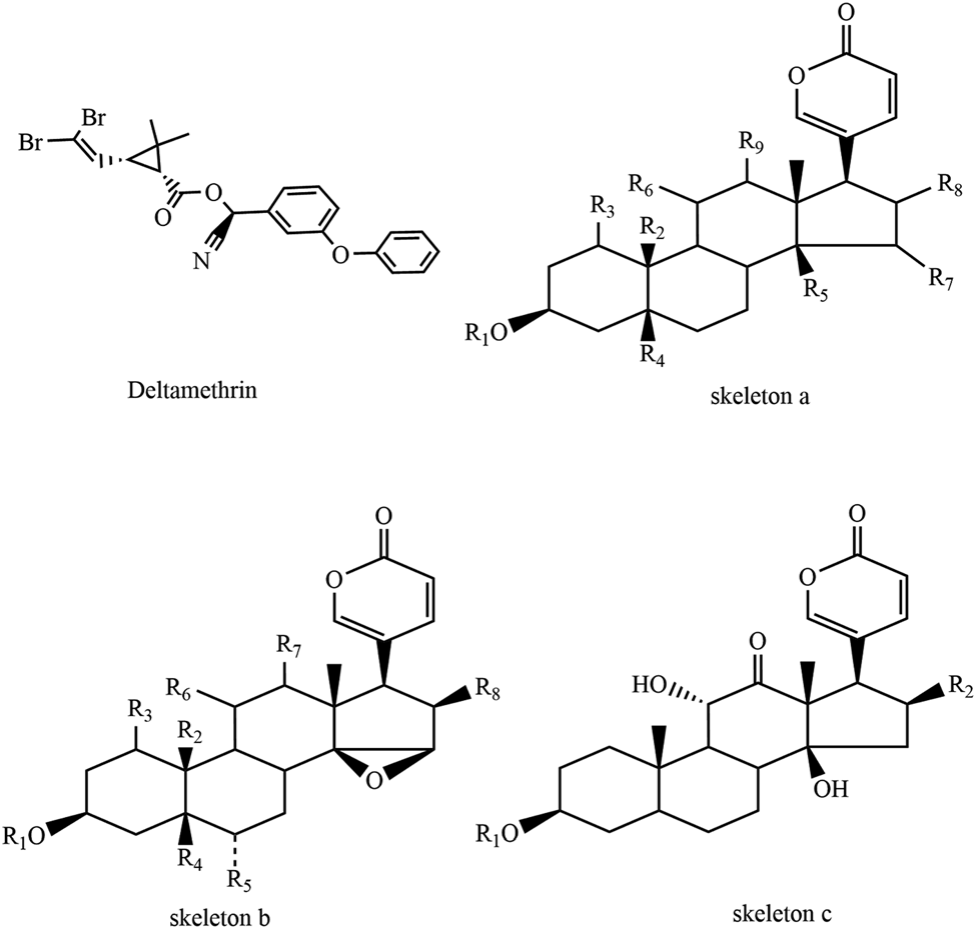
Skeletons of bufadienolides and chemical structure of deltamethrin.

Aquatic organisms are exposed to pesticides by the consumption of contaminated food and water, inhalation of pesticide residues or the absorption of pesticides through their skin. Synthetic pyrethroids are commonly used pesticides in agricultural activities; they are considered to pose minimal risk to human health,^7^ and its insecticidal activity has relatively low mammalian toxicity and extremely fast biodegradability. Deltamethrin (Fig. 1), a synthetic pyrethroid type II (produces even longer delay in sodium channel inactivation, leading to a persistent depolarization of the nerve membrane without repetitive discharge^8^), is highly effective against a broad spectrum of insects. Agricultural use of synthetic pyrethroids including deltamethrin has mainly resulted in the discharge of residues into drifts and runoffs,^9^ which ultimately binds rapidly to plants, sediments and organic matters in aquatic ecosystems.

Toads are amphibians that live both on land and in water. *Bufo bufo gargarizans* Cantor, the main source of Ch'an Su (an important traditional Chinese medicine and a primary component of Chinese patent drugs ‘Shexiang bao xin wan’ and ‘Liu shen wan’), are common amphibian species widely distributed in China. The population amount of *B. bufo gargarizans* Cantor has been sharply decreasing in recent years, which is greatly in contrast to the population of Australia's cane toads. They move so slowly that they could not escape predators immediately. Thus, they secret a variety of substances such as bufadienolides (Table 1) and peptide stored in granular secretory glands on their skin to defend themselves when attacked. This helps toads defend against or kill predators and sustain their lives.

**Table 1:** Chemical structures of bufadienolides in toad venom

Trivial name	Skeleton	Substituent group
Bufalin	a	R_1_=R_3_=H, R_2_=CH_3_, R_5_=OH, R_4_=R_6_=R_7_=R_8_=R_9_=H
Bufotalin	a	R_1_=R_3_=R_4_=R_6_=R_7_=R_9_=H, R_2_=CH_3_, R_5_=OH, R_8_=β-OAc
Desacetylbufotalin	a	R_1_=R_3_=R_4_=R_6_=R_7_=R_9_=H, R_2_=CH_3_, R_5_=OH, R_8_=β-OH
Resibufogenin	b	R_1_=R_3_=R_4_=R_5_=R_6_=R_7_=R_8_=H, R_2_=CH_3_
Cinobufagin	b	R_1_=R_3_=R_4_=R_5_=R_6_=R_7_=H, R_2_=CH_3_, R_8_=β-OAc
Cinobufotalin	b	R_1_=R_3_=R_5_=R_6_=R_7_=H, R_2_=CH_3_, R_4_=OH, R_8_=β-OAc
Arenobufagin	c	R_1_=R_2_=H

Toads live in water till they complete their metamorphosis and are regarded as an important species for monitoring environment pollution. Due to the relative ease of skin permeability and effect of life cycles on land and in water, toads are extremely sensitive to environmental contaminants^6,10,11^ including deltamethrin. Deltamethrin could be readily absorbed orally, and permeate into their body *via* the epithelial route. It is reported that^10^ pesticide contamination has negative effects on the growth of tadpoles, and chronic exposure at sub-lethal concentrations does not result in increased mortality or impaired growth. It has been proposed that persistent exposure to pesticides may be one of the factors contributing to the decline in the population of toads by affecting their growth and metabolism, immune system regulation and blood cell process.^12^

Many studies have shown the effects of environmental pollution on the death of tadpoles, frogs and toads. However, the influences of insecticide pollution on the venom secreted by the parotid gland in toads have rarely been studied. Herein, we hypothesize that deltamethrin contamination might influence the biosynthesis of toxic bufadienolides in toad venom. We evaluated the influences of cutaneous exposure to deltamethrin on the bufadienolide profiles in *B. bufo gargarizans* Cantor using an HPLC-triple quadrupole mass spectrometer in this study.

## 2. Methods

### 2.1 Materials and samples

*Bufo bufo gargarizans* Cantor specimens (caught in Nanjing, Jiangsu Province) were weighed and divided into four groups (*n* = 10). There was no significant difference between the groups. Toads in each group were fed in a semi-static system, where water and the toxicant were fully replaced every three days. The concentrations of deltamethrin (purchased from Bayer Healthcare) were 0.5 g L^−1^, 0.75 g L^−1^ and 1.0 g L^−1^. The weight, locomotor activity, appetite and exuviate of toads were observed and recorded. Fresh toad venom was collected by simply nipping the secretion gland with tweezers in adhesive tapes before and after exposure to deltamethrin. The venom samples were freeze-dried for further use.

### 2.2 Preparation of sample solutions

All of the dried toad venom samples were further pulverized to homogeneous powder. The powdered sample (2 mg) was taken in a 2 mL glass tube, and 1 mL methanol (HPLC-grade) was added to it. The accurate concentration of the sample was 1 mg mL^−1^. After covering the glass tubes with a cling film, ultrasonication was performed twice for 30 min. After centrifugation (12 000 rpm, 30 min), the supernatant was removed as sample solutions.

### 2.3 LC and MS conditions

HPLC analysis was performed using a Waters Acquity UPLC system (Waters, Milford, MA, USA) equipped with a Waters Xevo TQ tandem quadrupole mass spectrometer (Micromass MS Technologies, Manchester, UK). The separation of samples was achieved on a Synergi 2.5 μm Fusion C_18_ column (2.0 × 50 mm; Phenomenex, USA) with the column temperature maintained at 35 °C. A gradient elution of solvent A (ultra-pure water containing 0.1% formic acid) and solvent B (acetonitrile) was applied as follows: 0–2 min, 20–30% B; 2–6 min, 30–35% B; 6–9 min, 35–60% B; 9–10 min, 60–95% B; 10–11 min, 95% B; 11–11.2 min, 95–20% B, 11.2–12 min, 20% B. The flow rate of elution was 0.4 mL min^−1^ and the injected sample volume was set at 2 μL.

Two pairs of daughter ions that had stronger response and smaller interference were used for qualitative analysis by the prediction of UPLC-MS/MS. These ions are 385/367 and 349 for resibufogenin, 387/255 and 351 for bufalin, 403/349 and 367 for desacetylbufotalin, 417/399 and 371 for arenobufagin, 443/365 and 347 for cinobufagin, 445/349 and 367 for bufotalin, and 459/363 and 381 for cinobufotalin. The collision energy was set at 50 eV by screening ion pairs of target compounds, which was favorable for the determination of bufadienolides in toad venom. Ten individual biological samples were classified as an analytical batch. Quality control (QC) samples derived from a single QC pool and blank samples were analysed at the beginning and end of each batch.

### 2.4 Statistical analysis

The results were expressed as mean ± SEM. Statistical analysis was performed using two-tailed unpaired Student's *t*-test. A *p* value of less than 0.05 was considered statistically significant and a *p* value less than 0.01 was considered very significant.

## 3. Results

### 3.1 Mass spectrometric detection of bufadienolides in toad venom

**Fig. 2:**
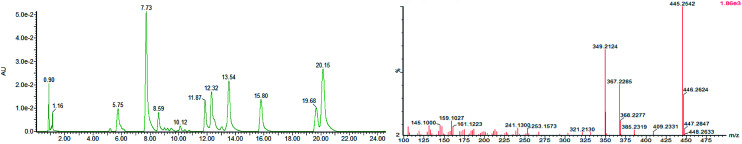
(Left) Total ion current (TIC) chromatogram of Venenum Bufonis (Chansu) by UPLC-MS. (Right) The MS/MS spectrum of bufotalin for the [M + H]^+^ ion at *m*/*z* 445, [M + H–HOAc]^+^ ion at *m*/*z* 385, [M + H–HOAc–H_2_O]^+^ ion at *m*/*z* 367 and [M + H–HOAc–3H_2_O]^+^ ion at *m*/*z* 349.

We detected small molecules in toad venom by Synapt Q-TOF-MS (Waters Corporation, Milford, MA) in positive ion mode. The total ion current (TIC) of the chemical constituents present in toad venom is shown in Fig. 2 (left). There were several types of constituents observed in toad venom, including steroids, bufotenines and other endogenous metabolites. Among these, most were steroid compounds named bufadienolides. Bufadienolides have a steroid nucleus with a six-membered lactone ring with hydroxyl group, aldehyde group, epoxy group, ethanoyl group and so on. They produced regular pyrolysis characteristics in the MS fragmentation. The dominant MS/MS fragments of bufadienolides are consistent with the results obtained in a previous study,^13^ including the loss of H_2_O and CO molecules, the initial loss of 42 Da (CH_2_CO) or 60 Da (HOAc), and the loss of 96 Da (d-pyrone ring). For instance, the [M + H]^+^ ion at *m*/*z* 401.2313 was identified as the bufotalin (Fig. 2, right). Its MS^2^ spectra included the following ions: [M + H–HOAc]^+^ ion at *m*/*z* 385, [M + H–HOAc–H_2_O]^+^ ion at *m*/*z* 367 and [M + H–HOAc–3H_2_O]^+^ ion at *m*/*z* 349. Thus, bufadienolides were characterized according to their precise monoisotopic mass, a minimum of three product ions in the MS/MS spectra, and the retention times against standards.

### 3.2 Development of quantitative LC-MS-MRM for bufadienolides in toad venoms

The Triple Quadrupole LC/MS system was used for the quantification of bufadienolides in toad venoms. External calibrations were performed for seven analytes by the least-squares linear regression method. The results confirmed that the assay was linear over the tested ranges (Table 2). The linear values ranged from 8 to 1000 ng mL^−1^. The LOD ranged from 1.51 to 26.14 ng mL^−1^, and the LOQ ranged from 5.03 to 87.14 ng mL^−1^ in the present study. The recovery of several bufadienolides were 95.1 to 112.8% (*n* = 3). The assay for the standard compounds was effectively performed, showing good linearity, recovery, sensitivity and repeatability.

**Table 2:** The standard curve, sensitivity precision, stability and accuracy of seven bufadienolide compounds detected by MRM (*n* = 6)

Analytes	Calibration curves	*r* ^2^	Linear range (ng mL^−1^)	LOD (ng mL^−1^)	LOQ (ng mL^−1^)	Precision (RSD, %)		Repeatability (RSD, %)	Stability (RSD, %)	Recovery (%), (2000 ng mL^−1^)	
						Intra-day	Inter-day	S1	S1	RSD	Mean
Resibufogenin	*y* = 390.54*x* − 11 595	0.9981	40–500	6.86	22.86	4.7	4.1	3.1	8.4	4.7	99
Desacetylcinobufotalin	*y* = 323.38*x* − 2644.9	0.9996	100–1000	26.14	87.14	6.0	5.9	5.8	4.6	3.8	102.2
Arenobufagin	*y* = 116.51*x* − 3166.7	0.9922	8–1000	1.53	5.10	7.3	3.8	5.8	3.7	7.1	103.8
Cinobufagin	*y* = 1062.9*x* −3985.9	0.9997	40–1000	6.32	21.05	8.8	6.8	4.2	6.1	7.5	102.3
Bufotalin	*y* = 1870.7*x* − 18 999	0.9998	40–1000	2.61	8.70	6.5	6.5	2.6	3.7	9.2	106.8
Cinobufotalin	*y* = 1815.2*x* − 13 523	0.9979	40–1000	1.74	5.80	4.8	5.5	5.7	5.8	6.3	95.1
Bufalin	*y* = 1088.8*x* − 18 181	0.9999	40–1000	1.51	5.03	—	—	5.2	5.8	4.4	112.8

### 3.3 Effects of deltamethrin cutaneous exposure on *Bufo bufo gargarizans* Cantor's survival rate and secretion of toad venom

Toads were exposed to different concentrations of deltamethrin for one month in a semi-static system. The loss in appetite of the highest concentration group was the highest. With the increase in time of exposure to deltamethrin, the activity of toads decreased significantly and their movements became stiff. It was observed that deltamethrin cutaneous exposure has no effects on the toad body weight (Table 3). However, toads cutaneously exposed to high dose of deltamethrin secreted less amount of venom, did not exuviate and had low survival rate in contrast to the normal toads.

**Table 3:** Weight of toads and venom secreted by toads before and after exposure to different concentrations of deltamethrin

Groups	Dose (g L^−1^)	Weight of toads (g)	Weight of venom (mg)
Normal		66.8 ± 16.3	1.5 ± 0.8
Deltamethrin	0.5	66.8 ± 13.5	1.25 ± 0.75
	0.75	71.3 ± 14.2	1.25 ± 0.63
	1.0	73.9 ± 13.9	1.0 ± 0.5

### 3.4 Effects of different concentrations of deltamethrin on levels of bufadienolides in toad venom secreted by *Bufo bufo gargarizans* Cantor

**Fig. 3:**
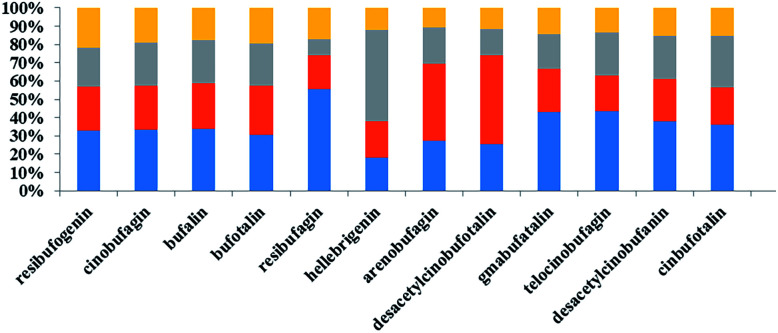
The percentages of bufadienolides in venom severed by normal and deltamethrin-exposed toads. Stacked bar graphs represent the percentages of bufadienolides in samples that were normal (blue), exposed to low dose of deltamethrin (orange), medium dose of deltamethrin (gray), and high dose of deltamethrin (yellow) groups.

As shown in Table 4, we observed that the relative content of 12 bufadienolides in toad venom markedly decreased after exposure to deltamethrin for one month. In the high dose deltamethrin (1.0 g L^−1^) group, the bufadienolide contents dropped by 1.5–3.2 fold. The low and middle dose of deltamethrin (0.5 and 0.75 g L^−1^) exposure slightly induced a decrease in bufadienolide content in toad venoms (Fig. 3). Among these constituents, bufalin in all polluted groups showed a significant decrease in comparison with the normal group (*p* < 0.05). The ratio of high-dose group and control group of telocinobufagin was 0.37, indicating a sharp decrease. Furthermore, we found that deltamethrin contamination had greater influence on the levels of low fat-soluble bufadienolides (desacetylcinobufotalin, gamabufotalin, telocinobufagin, desacetylcinobufagin, and cinobufotalin) than high fat-soluble ones (resibufogenin, cinobufagin and bufalin) in toad venoms. Therefore, deltamethrin contamination for one month could significantly change the expression pattern of toad's toxicant and reduced the contents of toxic bufadienolides in toad venoms.

**Table 4:** The relative level of bufadienolides in toad venom secreted by deltamethrin-exposed toads and normal toads (mean ± SEM; *n* = 5–7)^a^

Group	Dose (g L^−1^)	Resibufogenin	Cinobufagin	Bufalin	Bufotalin	Resibufagin	Hellebrigenin	Arenobufagin	Desacetylcinobufotalin	Gamabufotalin	Telocinobufagin	Desacetylcinobufagin	Cinobufotalin
Control		8.35 × 10^6^ ± 5.01 × 10^6^	4.55 × 10^7^ ± 1.47 × 10^7^	6.07 × 10^7^ ± 1.62 × 10^7^	8.45 × 10^7^ ± 1.23 × 10^7^	5.67 × 10^5^ ± 6.42 × 10^5^	7.62 × 10^3^ ± 4.49 × 10^3^	3.48 × 10^5^ ± 1.28 × 10^5^	2.34 × 10^5^ ± 1.31 × 10^5^	3.90 × 10^5^ ± 4.16 × 10^5^	6.68 × 10^5^ ± 7.19 × 10^5^	5.46 × 10^5^ ± 2.82 × 10^5^	4.70 × 10^6^ ± 3.00 × 10^6^
Deltamethrin	0.5	6.11 × 10^6^ ± 1.90 × 10^6^	3.29 × 10^7^ ± 8.12 × 10^6^	4.41 × 10^7^ ± 9.08 × 10^6^*	7.31 × 10^7^ ± 1.63 × 10^7^	1.88 × 10^5^ ± 1.30 × 10^5^	8.28 × 10^3^ ± 6.78 × 10^3^	5.37 × 10^5^ ± 5.27 × 10^5^	4.45 × 10^5^ ± 5.74 × 10^5^	2.15 × 10^5^ ± 1.45 × 10^5^	2.99 × 10^5^ ± 1.52 × 10^5^	3.36 × 10^5^ ± 1.73 × 10^5^	2.65 × 10^6^ ± 1.59 × 10^6^
	0.75	5.41 × 10^6^ ± 2.85 × 10^6^	3.20 × 10^7^ ± 8.30 × 10^6^	4.20 × 10^7^ ± 1.23 × 10^7^*	6.40 × 10^7^ ± 1.18 × 10^7^*	9.25 × 10^4^ ± 4.83 × 10^4^	2.07 × 10^4^ ± 2.11 × 10^4^	2.52 × 10^5^ ± 1.65 × 10^5^	1.30 × 10^5^ ± 1.23 × 10^5^	1.72 × 10^5^ ± 6.58 × 10^4^	3.61 × 10^5^ ± 1.22 × 10^5^	3.38 × 10^5^ ± 1.74 × 10^5^	3.66 × 10^6^ ± 1.79 × 10^6^
	1.0	5.51 × 10^6^ ± 3.16 × 10^6^	2.59 × 10^7^ ± 9.95 × 10^6^*	3.16 × 10^7^ ± 1.22 × 10^7^**	5.28 × 10^7^ ± 1.58 × 10^7^**	1.73 × 10^5^ ± 2.13 × 10^5^	5.09 × 10^3^ ± 6.23 × 10^3^	1.34 × 10^5^ ± 5.27 × 10^4^**	1.05 × 10^5^ ± 7.14 × 10^4^	1.28 × 10^5^ ± 1.27 × 10^5^	2.10 × 10^5^ ± 1.43 × 10^5^	2.18 × 10^5^ ± 1.08 × 10^5^	1.99 × 10^6^ ± 1.09 × 10^6^

a *P < 0.05, **P < 0.01, compared with control.

## 4. Discussion

Agricultural use of synthetic pyrethroids including deltamethrin has mainly resulted in the presence of residues in drifts and runoffs, which ultimately bind rapidly to plants, sediments and organic matters in aquatic ecosystems. Toads also live in water and could easily access deltamethrin. They spawn in water and their descendants live in water until metamorphosis is completed, and thus have high probability of exposure to deltamethrin contamination. Our research showed that toads exposed to high concentration of deltamethrin survive the least, do not exuviate, and their movements are stiff. This may relate to the neurotoxicity of pyrethroids. Pyrethroids are ion channel toxins that interfere with the function of the nervous system. The majority of insecticides sold are neurotoxic, either inhibiting neurotransmitters or affecting voltage-gated sodium channels, which are located along the cell membrane on the neuron tail of axons. At higher concentrations, pyrethroids may also interfere with the chloride channels.^14^ Upon exposure to pyrethroids, the sodium channels malfunction and remain open instead of returning to a closed state after the initiation of the action potential, which leads to repetitive firing or depolarization that causes tremors or involuntary movements.^14^ Pyrethroids are also shown to interact with the γ-amino butyric acid (GABA) receptor–ionophore complex to cause neurotoxicity^15^ and cause an imbalance and affect movement indirectly *via* inhibiting ATPases associated with active transport enzymes such as Na^+^–K^+^–ATPase and Ca^2+^–Mg^2+^–ATPase.^14,16^ The neurotoxicity of deltamethrin is thought to affect the central system mainly, or at least originates in higher nerve centers of the brain, which might cause damage to the toad's nervous system.

Moreover, for the first time, we report that the level of bufadienolides in venom drops after exposure to deltamethrin in this study. The changes in the toxin profiles might reduce the toad's defense power. The bufadienolide substance in venom plays a key role in the chemical defense of toads. First, bufadienolide toxins affect the physiological transport of ions. These constituents could regulate sodium and water homeostasis in the toad skin by inhibiting Na^+^- and K^+^-dependent adenosine triphosphatase (ATPase), an enzyme crucial to sodium and water homeostasis regulation.^17^ Moreover, these toxins, localized largely to toad parotid glands, can be expressed as a secretion during a predatory event. Bufadienolides are known to exert a cardiotoxic effect by inhibiting the cardiac sodium pump to kill the native predators. Moreover, the toad venom containing bufadienolides is a local irritant. It has been observed that the bufadienolide extracts had a local irritant action on the rabbit ear vein.^18^ Cardiotoxicity and irritation are very effective against predators whose limited prey-handling skills require considerable time to subdue their prey, and/or swallow their prey whole (*e.g.*, predatory bird, fish, lizards, snake, and large amphibians). It may act in concert with ingestion-delaying behaviour. These toxins are not only highly toxic to predators, but also could adversely affect the predator's taste due to the strong irritation, which are beneficial to the remaining toad population, possibly aided by predator learning. Therefore, the decrease in bufadienolide content in toads exposed to deltamethrin would inevitably decline their native defense power, and then lead to their death or make them more prone to be easily hunted by predators.

To date, the reasons for these observations were unknown. We inferred that deltamethrin might affect the biosynthesis of bufadienolides from cholesterol in venom. It has been shown that there were hundreds of proteins in toad venom, among which a variety of enzymes were found, including 4,3-ketosteroid-5-reductase, hydroxysteroid dehydrogenase, prolyl-hydroxylase, aldehyde dehydrogenase, lactate dehydrogenase A, neutral cholesterol ester hydrolase, hydroxyacyl-CoA dehydrogenase, (14)-sterol reductase, hydroxyacylglutathione hydrolase, and some CYP450 family enzymes.^19^ These enzymes have the ability to bio-transform various steroids by oxidation and reduction of hydroxyl groups, dehydration, removal of acetyl groups, and hydrolysis. It is possible that exposure to deltamethrin pesticides induces the changes in the expressions of these metabolic enzymes and further result in the decrease in bufadienolide contents in toad venom.

In addition, we noticed that the change in contents of two compounds after deltamethrin exposure was different from that of other bufadienolides. The reason for this was unknown. In plant science, many environmental stress factors (high UV, low temperature, pathogen attack and wound) could enhance the biosynthesis of secondary metabolites by plants to ensure their survival, persistence and competitiveness. Similarly, we speculated that exposure to low dose of deltamethrin might be a moderate stress factor and stimulate the defense response of toads to induce the accumulation of bufadienolides in venom. However, exposure to high dose of deltamethrin would produce adverse effects on the biosynthesis of bufadienolides in toads.

## 5. Conclusions

In this study, for the first time, we report that cutaneous exposure to high levels of deltamethrin pesticides could reduce the contents of defensive chemicals, namely, bufadienolides, in toad venom. This could reduce toads' defense power and make them easily hunted by predators, which is an underlying reason for the rapid decline in the population of *B. bufo gargarizans*. It also gave us some inspiration to explore other types of safe, less toxic and rapidly degradable insecticides to protect important and endangered species and maintain the ecosystem balance.

## Conflicts of interest

There are no conflicts to declare.

## Supplementary Material
